# A total infectome framework for resolving complex disease etiology in aquaculture

**DOI:** 10.1007/s44307-026-00125-8

**Published:** 2026-08-07

**Authors:** Yichun Xu, Hanlin Liu, Weichen Wu, Yuchao Gu, Naiyou Zhang, Cancan Zhang, Renjun Zhou, Defeng Zhang, Shaoping Weng, Mang Shi, Jianguo He, Jian He

**Affiliations:** 1 State Key Laboratory for Biocontrol, Southern Laboratory of Ocean Science and Engineering (Guangdong, Zhuhai), School of Marine Sciences, Sun Yat-Sen University, 135 Xingang Road West, Guangzhou, 510275 PR China; 2 School of Life Sciences, Sun Yat-Sen University, 135 Xingang Road West, Guangzhou, 510275 PR China; 3 School of Medicine, Shenzhen Campus of Sun Yat-sen University, Sun Yat-sen University, Shenzhen, 518107 PR China; 4 Pearl River Fisheries Research Institute, Chinese Academy of Fishery Sciences, Guangzhou, 510380 PR China

**Keywords:** Total infectome, Etiology, Grass carp, Overwintering syndrome, Flavobacterium psychrophilum

## Abstract

Traditional pathogen isolation can establish disease causality but often fails in complex infections involving unknown or multiple microbial backgrounds, whereas metatranscriptomics comprehensively captures the total infectome without directly resolving the primary etiological agent. Here, using overwintering syndrome (OWS) in grass carp, a widespread but unresolved aquaculture disease in China, as a disease model, we developed an integrated strategy that links comparative total infectome analysis with targeted isolation, experimental infection, and post-challenge infectome validation. Multi-organ metatranscriptomic profiling of healthy and diseased grass carp identified 107 microorganisms spanning viruses, bacteria, and eukaryotic microbes. Comparative infectomics prioritized *Flavobacterium psychrophilum* through its consistent disease enrichment, lesion-site abundance, multi-organ dissemination, and recurrence across affected hosts. Infection with *F. psychrophilum* alone reproduced the clinical signs, tissue pathology, mortality patterns, and infectome signatures of natural OWS, establishing it as the primary etiological agent. Temperature-shift experiments further showed that disease progression is promoted by warming from low temperature conditions, explaining the seasonal emergence of OWS. This work resolves a major grass carp disease and provides a generalizable framework for converting unbiased pathogen discovery into causal inference in complex infectious disease systems.

**Supplementary Information:**

The online version contains supplementary material available at 10.1007/s44307-026-00125-8.

## Introduction

Identifying the causative agent of an infectious disease has traditionally relied on pathogen isolation, culture, histopathology, targeted molecular assays, and experimental infection (Fredricks and Relman 1996; Houpikian and Raoult 2002; Yang and Rothman 2004). These approaches remain the gold standard for establishing disease causality, particularly when a candidate pathogen can be isolated and shown to reproduce disease under controlled conditions. However, they are often difficult to apply when the etiological agent is unknown, unculturable, low-abundance, or embedded within a complex microbial community (Abdelsalam et al. 2023; Lipkin 2013). As a result, many emerging diseases remain unresolved despite extensive diagnostic efforts.

Metagenomic and metatranscriptomic sequencing have transformed pathogen discovery by enabling unbiased detection of microorganisms without prior assumptions (Chiu and Miller 2019; Ojala et al. 2023; Wilson et al. 2019). In particular, RNA-based metatranscriptomics provides a “total infectome” framework capable of simultaneously detecting RNA viruses, actively transcribing DNA viruses, bacteria, fungi, and other eukaryotic microorganisms within the same sample. Recent studies have demonstrated the utility of total infectome analysis for comprehensive characterization of microbial communities across diverse systems, including profiling pathogen spectra in human respiratory diseases, surveying microbial diversity in domestic animals, and monitoring pathogen diversity in wildlife reservoirs (Huang et al. 2022; Shi et al. 2022; Sun et al. 2024; Xin et al. 2025). Yet identifying microorganisms is not equivalent to identifying the cause of disease. Total infectome analyses frequently recover complex mixtures of commensal, environmental, opportunistic, secondary, and potentially pathogenic organisms, making etiological interpretation difficult. A comparative cohort design can help address this challenge by prioritizing candidate pathogens according to disease enrichment, recurrence across affected individuals, abundance, organ distribution, lesion-site localization, and consistency with pathological features (Huang et al. 2022; Li et al. 2019). When integrated with targeted isolation, experimental infection, re-isolation, and post-challenge infectome profiling, this strategy provides a coherent framework linking unbiased pathogen discovery to causal validation.

Fish diseases provide an important yet particularly challenging setting for applying this framework. Aquatic environments support extensive microbial diversity and facilitate pathogen persistence and transmission through water (Oidtmann et al. 2018), while intensive aquaculture practices, shared water systems, high stocking density, and seasonal environmental stress further increase disease risk (Ashley 2007; Canosa and Bertucci 2023; Kautsky et al. 2000; Raymo et al. 2024). Consequently, many disease outbreaks in aquaculture remain etiologically unresolved, particularly when affected fish contain complex microbial communities with multiple plausible pathogens (O’Halloran et al. 2022). Recent years have seen increasing reports of unexplained or polymicrobial disease syndromes in fish, highlighting the limitations of conventional culture- and target-based diagnostics for identifying novel or unexpected etiological agents (Abdelsalam et al. 2023; Kotob et al. 2016; Shafi 2025). One representative example is grass carp overwintering syndrome (OWS), an emerging disease affecting grass carp (*Ctenopharyngodon idella*), one of the world’s most economically important freshwater aquaculture species (FAO 2024). Since 2019, OWS has repeatedly occurred in China during late overwintering and early spring warming periods, causing lethargy, reduced feeding, skin ulceration, caudal muscle hemorrhage, and high mortality (Feng et al. 2026b; Huang et al. 2020). Despite its expanding geographic distribution and substantial economic impact, the primary cause of OWS has remained unresolved, likely because diseased fish harbor complex microbial communities and multiple candidate pathogens.

Here, we used grass carp OWS as a case study to test an integrated strategy combining cohort-based total infectome analysis with classical pathogen validation. We performed multi-organ metatranscriptomic profiling of healthy and diseased grass carp to comprehensively characterize the viral, bacterial, fungal, and eukaryotic microorganisms associated with OWS. By integrating differential abundance, cross-individual recurrence, organ distribution, and lesion-site enrichment, we prioritized *F. psychrophilum* as the leading etiological candidate. We then isolated this bacterium from diseased tissue and validated its causal role through experimental infection, re-isolation, histopathology, and post-challenge total infectome analysis. Finally, temperature-dependent infection experiments explained the seasonal emergence of OWS during early spring warming. Together, this study resolves the etiology of grass carp OWS and establishes a generalizable framework for identifying causative agents in complex aquatic disease syndromes.

## Results

### Grass carp overwintering syndrome exhibits expanding outbreaks and complex infectious features

**Fig. 1:**
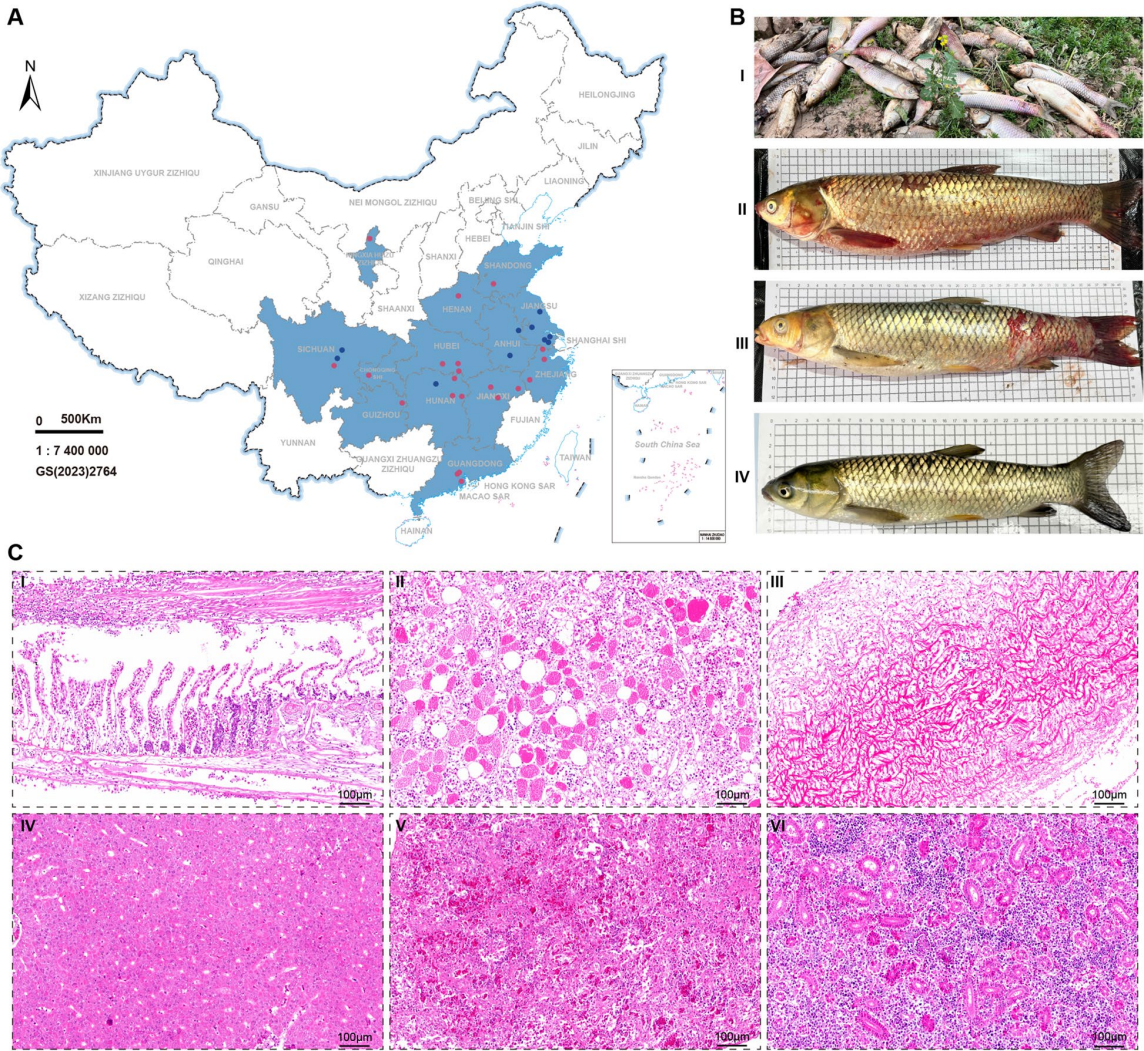
Epidemiological survey, representative clinical signs, and histopathological observations of grass carp overwintering syndrome. **A** Geographic distribution of reported grass carp overwintering syndrome (OWS) cases (blue points) and sampling sites included in this study (red points). **B** Representative grass carp showing typical clinical signs of OWS (I–III) and healthy individuals (IV). **C** Histopathologic lesions in diseased grass carp. I. Sloughing of the gill filaments. II. Myofiber lysis with marked vacuolation. III. Disorganization of the skin structure with rupture of dermal collagen fibers. IV-VI. The liver, spleen, and kidney remained largely intact, with no obvious histopathological lesions. Scale bars = 100 μm

Grass carp OWS was first documented in 2019 at multiple aquaculture areas within the Yangtze River basin (Huang et al. 2020). To assess its current distribution, we conducted epidemiological surveys from 2021 to 2025 across major grass carp-producing regions in China. OWS cases were detected not only in the Yangtze River basin, including Zhejiang, Jiangxi, and Chongqing, but also in the Pearl River basin and the Yellow River basin, indicating a broader cross-basin distribution (Fig. 1A).

Diseased fish exhibited typical OWS-associated clinical manifestations, including external hemorrhages, subcutaneous muscle ulceration, reddening of the head, and swelling and erythema of the snout (Fig. 1B). These cases occurred mainly during the late overwintering and early spring warming period.

Histopathological examination showed prominent lesions in external and barrier tissues. Diseased fish exhibited sloughing of gill filament, extensive muscle fiber necrosis and degeneration in skeletal muscle, and disruption of skin architecture with rupture of dermal collagen fibres (Fig. 1C). In contrast, the liver, spleen, and kidney remained largely intact, with no obvious histopathological lesions.

### Total infectome analysis reveals a highly complex pathogen landscape in OWS

To characterize the microbial landscape associated with OWS, we performed total RNA-based metatranscriptomic sequencing on eight organs from healthy and diseased grass carp sampled from different aquaculture farms in China (Fig. S1A). Each organ was processed as an independent library, generating 80 metatranscriptomes in total. After quality filtering and rRNA depletion, 8.09 billion high-quality non-rRNA reads were retained, with an average of 101.2 million reads per library, providing sufficient sequencing depth for broad pathogen detection (Destras et al. 2026) (Fig. S1B). Host identity was confirmed by COI-5P sequence analysis (Fig. S1C).

**Fig. 2:**
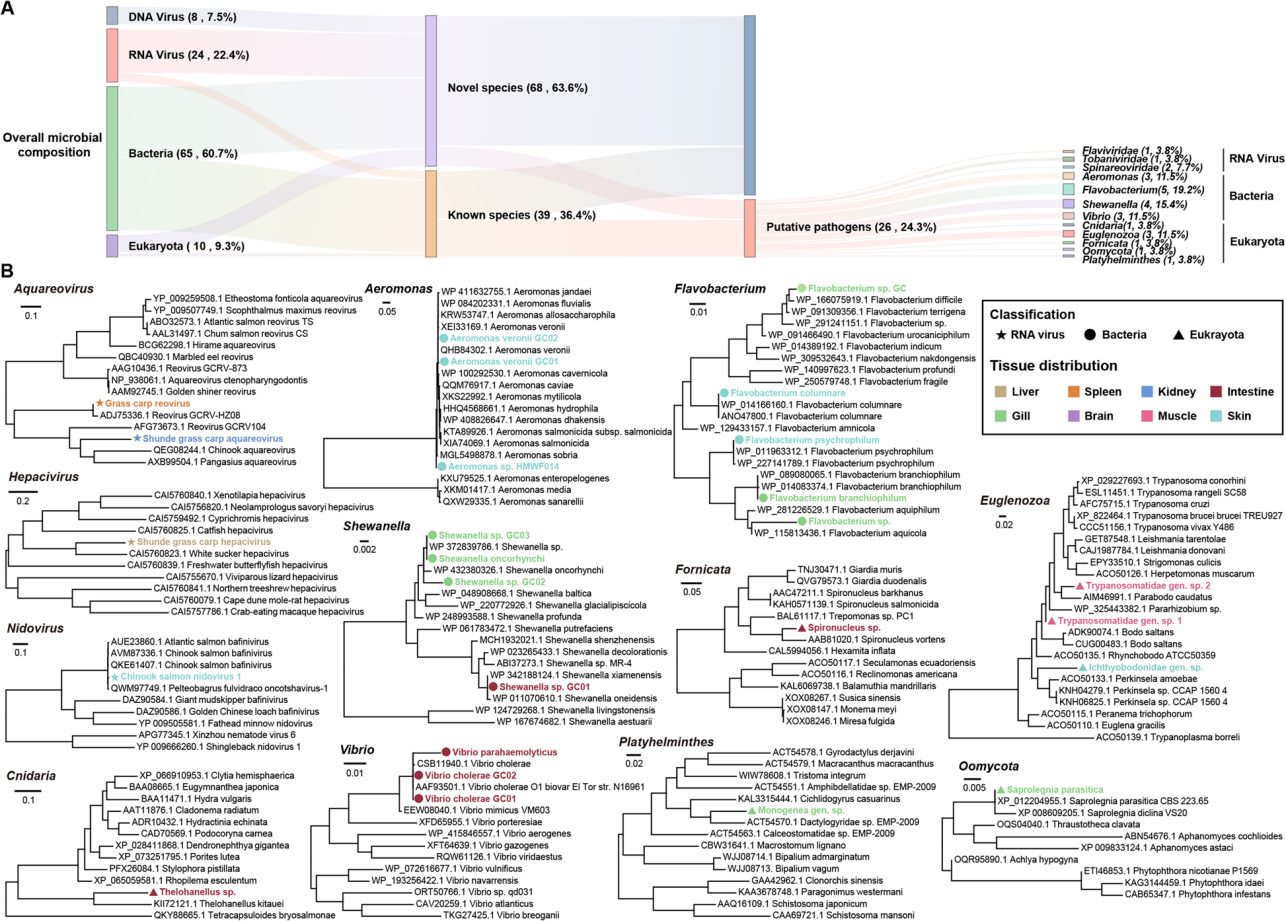
Overview of the total infectome. **A** Composition of detected microorganisms in grass carp. **B** Phylogenetic trees of microorganisms known to be associated with fish diseases, including viruses, bacteria, and eukaryotes. Trees were estimated using the maximum likelihood method based on the RdRp protein for RNA viruses, the beta-subunit of RNA polymerase (rpoB) protein for bacteria, and elongation factor 1 alpha (EF1α) for eukaryotes

Across these libraries, we identified 107 dominant microbial species, including 32 viruses, 65 bacteria and 10 eukaryotic microorganisms. These taxa spanned 9 RNA viral supergroups, 2 DNA viral families, 7 bacterial phyla, and 7 eukaryotic phyla, demonstrating a diverse infectome in grass carp (Fig. S2). Bacteria constituted the largest fraction of detected microorganisms (60.7%), followed by RNA viruses (22.4%), eukaryotes (9.3%), and DNA viruses (7.5%). Notably, 68 species (63.6%) were putative novel species, indicating that a substantial proportion of the grass carp-associated infectome remains previously uncharacterized (Fig. 2A).

Using a pathogen-relevance framework that included known pathogens, opportunistic pathogens and uncharacterized lineages related to vertebrate-infecting pathogens, we identified multiple microorganisms with potential disease relevance (Huang et al. 2022; Sun et al. 2024) These include 4 RNA viruses, 15 bacterial species, and 7 eukaryotic taxa (Fig. 2B). Among them were viral lineages from *Flaviviridae*, *Tobaniviridae* and *Spinareoviridae*, bacterial genera commonly associated with aquatic animal disease, including *Aeromonas*, *Flavobacterium*, *Shewanella* and *Vibrio*, and several parasitic eukaryotes from Cnidaria, Euglenozoa, Fornicata and Platyhelminthes.

Several detected agents expanded the known pathogen diversity of grass carp. These included grass carp hepacivirus, representing the first hepacivirus detected in grass carp; Chinook salmon nidovirus 1, previously reported from salmonids; and Shunde grass carp aquareovirus, a divergent aquareovirus with low sequence similarity to known grass carp reoviruses. Together, these results show that OWS-affected grass carp harbour a complex mixture of known, opportunistic and previously undescribed microorganisms, underscoring the need to distinguish disease-associated agents from background or secondary members of the infectome.

### Comparative infectomics prioritizes *F. psychrophilum* as the dominant etiological candidate

**Fig. 3:**
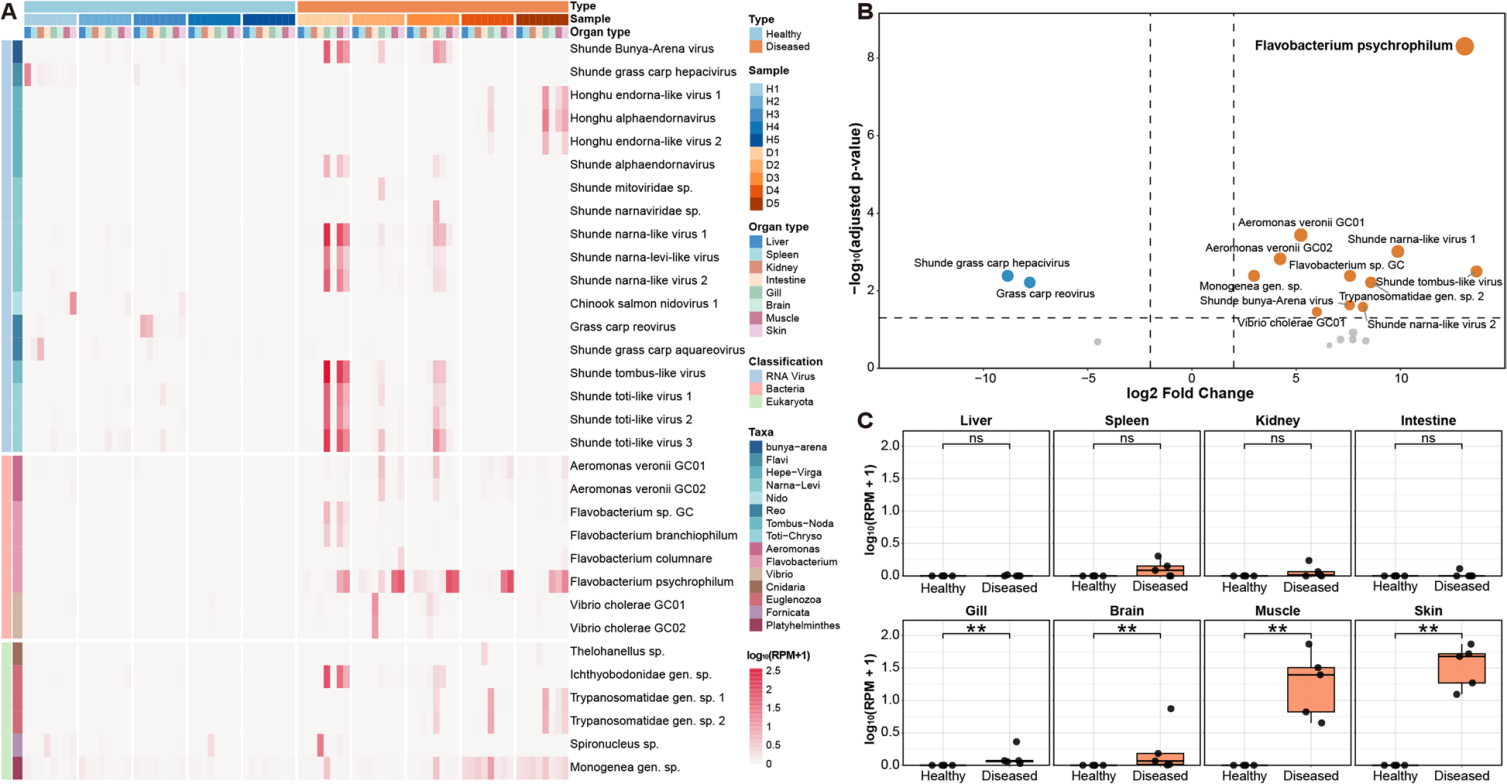
Distribution and differential abundance of microorganisms in grass carp. **A** Distribution and abundance of microorganisms across different organs. Relative abundance was normalized as reads per million total reads (RPM), and only microorganisms with RPM > 1 are shown to minimize potential contamination. **B** Volcano plot showing differential microbial abundance between diseased and healthy grass carp, including bacteria, eukaryotes, and viruses. Each point represents a microbial species, with colors indicating upregulated (orange), downregulated (blue), or non-significant changes (gray). Differential abundance was defined as |log₂FC|≥ 2 and FDR < 0.05. **C** Organ tropism of F. psychrophilum across eight grass carp organs, visualized using log_10_(RPM +1)-transformed abundance values. Statistical differences in F. psychrophilum abundance between healthy and diseased fish were assessed using the Wilcoxon rank-sum test. ns, not significant (*P* ≥ 0.05); *P < 0.05; **P < 0.01

To identify microorganisms most strongly associated with OWS, we compared microbial abundance profiles between diseased and healthy grass carp. Using an abundance threshold of RPM ≥ 1 (mapped reads per million non-rRNA reads), a total of 33 microbial taxa were retained for quantitative analysis, including RNA viruses, bacteria and eukaryotic microorganisms (Fig. 3A). Overall microbial profiles separated diseased fish from healthy controls, with diseased fish showing higher microbial abundance and broader taxonomic representation.

Differential abundance analysis identified several taxa enriched in diseased fish (Fig. 3B). Among these, *F. psychrophilum* exhibited the strongest and most consistent disease-associated signal. It was detected across all diseased individuals and multiple organs, whereas most other microbial taxa showed sporadic occurrence or were restricted to individual samples. Organ-level analysis further showed that *F. psychrophilum* reached its highest abundance in muscle and skin, corresponding to the major lesion sites of OWS (Fig. 3C).

Several eukaryotic microorganisms, including *Ichthyobodonidae gen.* sp., *Trypanosomatidae gen.* sp. 1, *Trypanosomatidae gen.* sp. 2 and *Thelohanellus* sp., were also enriched in diseased fish, but their detection was less consistent and lacked the lesion-associated distribution observed for *F. psychrophilum*, suggesting that they are more likely to represent opportunistic or secondary infections rather than the primary etiological agent. Viral taxa from the Bunya-Arena, Narna, Tombus-Noda and Toti supergroups were likewise increased in diseased samples. However, phylogenetic analysis placed these viruses within invertebrate-associated lineages rather than established vertebrate-infecting clades (Fig. S3A). Their abundance profiles also closely paralleled that of *Ichthyobodonidae gen.* sp., suggesting that they were more likely associated with parasitic eukaryotes than directly infecting grass carp (Fig. S3B).

Together, the convergence of disease enrichment, cross-individual recurrence, multi-organ dissemination and lesion-site abundance prioritized *F. psychrophilum* as the leading etiological candidate for grass carp OWS.

### Experimental infection recapitulates OWS pathology and infectome signatures

**Fig. 4:**
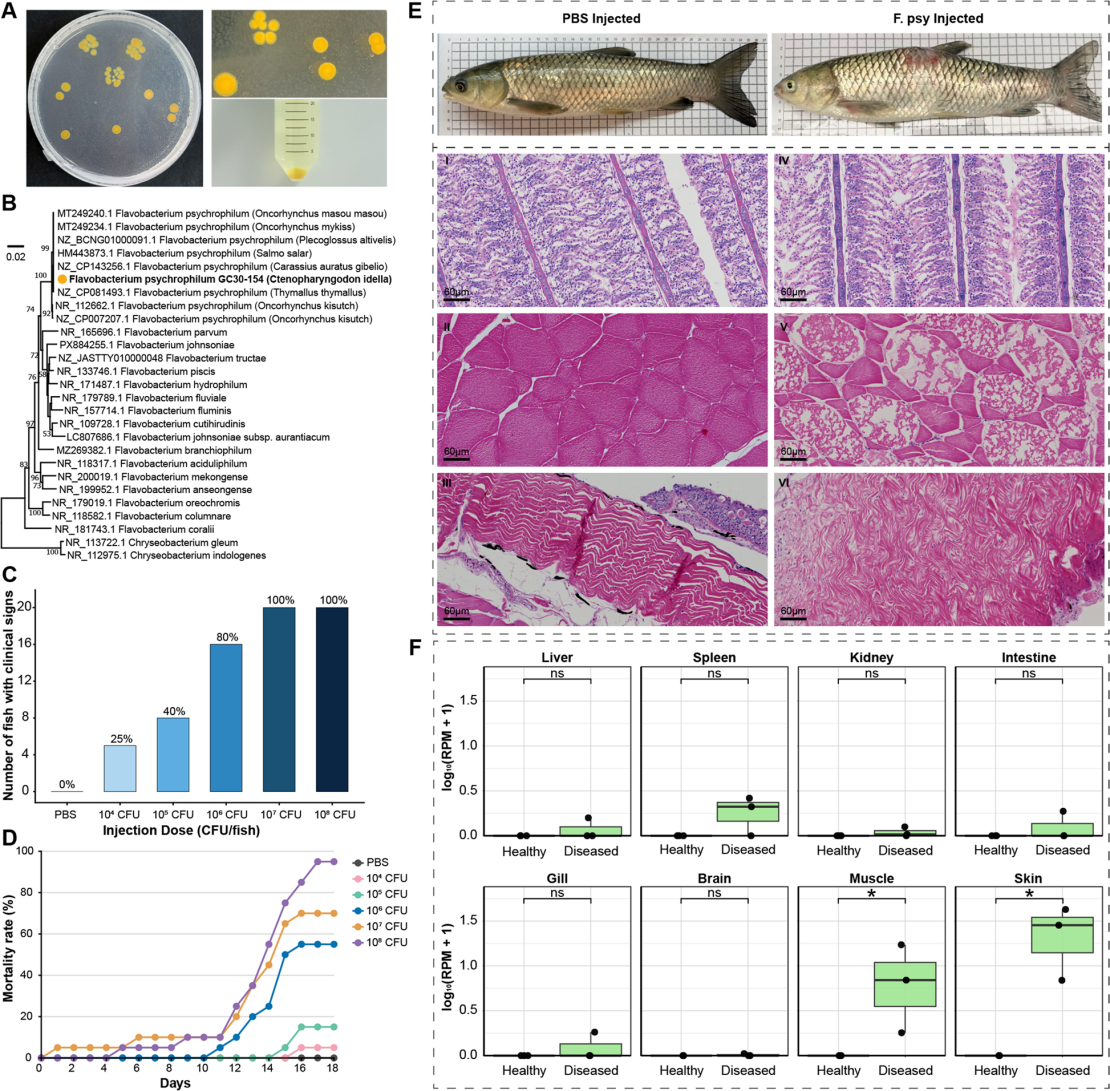
Isolation and identification of *F. psychrophilum*, experimental infection, and post-challenge total infectome analysis. **A** Colony morphology of *F. psychrophilum* on TYES solid medium (left) and in TYES liquid medium (right), showing characteristic yellow colonies. **B** Neighbor-joining phylogenetic tree based on 16S rRNA gene sequences, with bootstrap values indicated at nodes, showing that the isolate *F. psychrophilum* GC30-154 belongs to the *F. psychrophilum* clade with strong support (bootstrap = 1000). **C** Occurrence of clinical signs in grass carp following injection with different doses of F. psychrophilum. Bars indicate the number of fish showing clinical signs in each group, and percentages above the bars indicate the corresponding morbidity. **D** At a constant temperature of 15 °C, mortality curves of grass carp following experimental infection with different doses of *F. psychrophilum,* with PBS-treated fish as controls. **E** Clinical signs and histopathological changes in experimentally infected fish and PBS-injected controls. Gross lesions included focal erythema and swelling at the dorsal injection site, reddening around the pectoral-fin base, mild snout reddening, and tail erosion. H&E-stained sections showed severe muscle fiber degeneration and vacuolation together with disorganization of skin structure in infected fish, whereas no obvious pathological changes were observed in the gills of either infected or control fish. Scale bars = 60 μm. **F** Comparison of F. psychrophilum abundance across different organs between experimentally infected fish and PBS-injected control fish. In the figure, "Diseased" and "Healthy" denote the experimentally infected and PBS-injected control groups, respectively. The y-axis indicates log_10_(RPM +1)-transformed abundance values. Statistical significance was assessed using the Wilcoxon rank-sum test. ns, not significant (*P* ≥ 0.05); *P < 0.05; **P < 0.01

To experimentally validate the pathogenic role of *F. psychrophilum*, bacteria were isolated from lesion-associated muscle tissues of grass carp with typical OWS signs. After incubation on TYES agar at 15 °C, morphologically uniform pale-yellow colonies were obtained (Fig. 4A). 16S rRNA gene sequencing and phylogenetic analysis placed the representative isolate, *F. psychrophilum* GC30-154, within the *F. psychrophilum* clade with strong bootstrap support (Fig. 4B).

Healthy grass carp were then challenged by intramuscular injection with graded doses of *F. psychrophilum*. PBS-injected controls showed no abnormal clinical signs or mortality throughout the experiment. In contrast, infected fish developed dose-dependent disease signs from day 4 post-injection, including local erythema and swelling at the injection site. Morbidity increased with bacterial dose, reaching 25%, 40%, 80% and 100% in the 10^4, 10^5, 10^6 and 10^7–10^8 CFU groups, respectively (Fig. 4C). Sustained mortality was observed in most groups starting around day 12 and increased in a dose-dependent manner thereafter, reaching 5%, 15%, 55%, 70% and 95% by day 18 in the 10^4 to 10^8 CFU groups, respectively (Fig. 4D). *F. psychrophilum* was re-isolated from lesion-associated muscle tissues of deceased fish (Fig. S4A).

Experimentally infected fish reproduced the major clinical and pathological features of natural OWS. Infected fish developed focal erythema and swelling at the dorsal injection site, reddening around the pectoral-fin base, mild snout reddening and tail erosion (Fig. 4E). Histopathological examination showed severe disruption of muscle fibre architecture, extensive vacuolar degeneration and disorganization of skin structure, whereas corresponding tissues from PBS-injected controls remained intact (Fig. 4E). These lesions closely resembled those observed in naturally diseased grass carp.

To determine whether the experimental disease was primarily driven by *F. psychrophilum* rather than secondary microorganisms, we further performed post-challenge total infectome analysis. Only *F. psychrophilum* was consistently detected at high abundance in experimentally infected fish, with strongest enrichment in muscle and skin, while PBS-injected controls showed no signal (Fig. 4F and S4B). This organ distribution recapitulated the pattern observed in naturally diseased fish (Fig. 4F). Together, these results demonstrate that *F. psychrophilum* is sufficient to reproduce the clinical signs, tissue pathology and infectome signatures of grass carp OWS.

### Disease progression is strongly temperature dependent

**Fig. 5:**
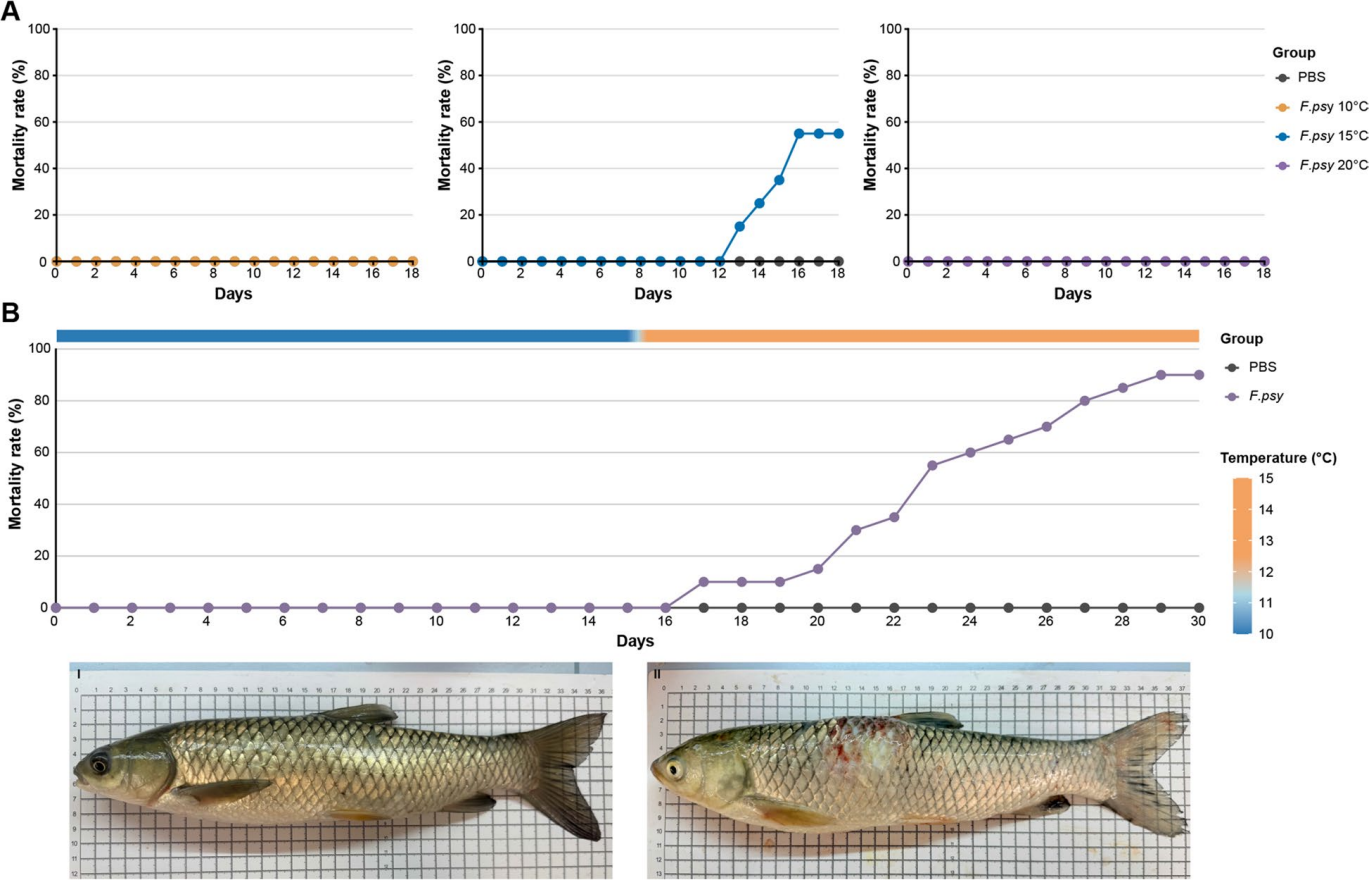
Temperature-dependent infection experiments of grass carp with *F. psychrophilum.* **A**. Mortality of grass carp challenged with *F. psychrophilum* under constant temperatures of 10 °C, 15 °C, and 20 °C. Fish mortality was monitored for 18 days. **B** Mortality under a gradual temperature increase regime. Fish were maintained at 10 °C for the first 15 days, then gradually increased to 15 °C, with the entire experiment lasting 30 days. Fish were intramuscularly injected into the dorsal musculature with 10^6 CFU/fish; PBS-injected fish served as controls

To assess whether water temperature influences disease outcome, grass carp were intramuscularly challenged with a fixed dose of *F. psychrophilum* (10^6 CFU/fish) and maintained at 10 °C, 15 °C, and 20 °C. Mortality differed markedly among temperature groups. Fish maintained at 15 °C exhibited the highest mortality (55%), whereas no death occurred in fish maintained at 10 °C or 20 °C during the observation period (Fig. 5A).

We next performed a temperature-shift experiment to mimic the overwintering-to-spring transition. After *F. psychrophilum* challenge, fish were first maintained at 10 °C for 15 days, during which no obvious clinical abnormalities or mortality was observed. The water temperature was then gradually increased to 15 °C. Following this temperature elevation, infected fish gradually developed OWS-like signs, including external ulcers, and mortality increased sharply, reaching 95% by day 14 after warming (Fig. 5B). PBS-injected controls remained healthy throughout the experiment.

These results show that the disease in grass carp caused by *F. psychrophilum* is strongly temperature dependent, with disease progression triggered most efficiently under conditions resembling the early spring warming period when natural OWS outbreaks occur.

## Discussion

The continued emergence and re-emergence of infectious diseases increasingly challenges conventional pathogen discovery frameworks, particularly in aquatic systems where hosts are continuously exposed to diverse environmental microorganisms (Hutson et al. 2023; Morens et al. 2004). In such settings, diseased individuals often harbor complex mixtures of viruses, bacteria, fungi, and parasites, making it difficult to distinguish the true etiological agent from secondary or background members of the infectome (Bogdanos et al. 2013; Huang et al. 2022; Kotob et al. 2016). Grass carp OWS represents a typical example of this problem. Despite its rapid spread across major aquaculture regions in China and its substantial economic impact, the causative agent of OWS has remained unresolved for years (Feng et al. 2026a; Huang et al. 2020). In this study, we combined epidemiological investigation, cohort-based total infectome analysis, targeted isolation, experimental infection, and post-challenge infectome profiling to systematically resolve the etiology of OWS. Through comparative infectomics, *F. psychrophilum* was identified as the only microorganism consistently enriched in diseased fish, broadly distributed across organs, and associated with lesion sites. Subsequent experimental validation confirmed *F. psychrophilum* as the primary etiological agent of OWS.

*F. psychrophilum* is classically recognized as a cold-water pathogen of salmonids, where it causes bacterial cold-water disease and rainbow trout fry syndrome (Nematollahi et al. 2003; Starliper 2011). However, the infection pattern observed here differs substantially from the classical salmonid-associated disease model (Barnes and Brown 2011). In grass carp, lesions were concentrated primarily in skin and muscle tissues rather than visceral organs such as the spleen and kidney (Deng et al. 2024, 2022; Madsen and Dalsgaard 1999), suggesting altered tissue tropism in this host. Moreover, disease progression showed a distinct thermal profile. Whereas classical outbreaks in salmonids are typically associated with temperatures below 10 °C (Hesami et al. 2011; Vaibarová and Čížek 2024), OWS-associated disease in grass carp developed most efficiently around 15 °C and was strongly exacerbated during warming from overwintering temperatures. Given the major physiological and ecological differences between salmonids and warm-water cyprinids (Liang et al. 2022), these findings suggest that the isolate identified here may represent a host-adapted ecological variant with altered temperature-dependent pathogenicity. More broadly, the increasing connectivity among freshwater aquaculture systems, including the rapid expansion of salmonid farming (Ma et al. 2025; Xiao et al. 2026) and widespread polyculture practices in China (Song and Zhang 2025), may facilitate transmission of pathogens across previously separated host systems, creating new opportunities for host shifts and disease emergence.

In addition to the primary etiological agent, total infectome analysis also identified several other microorganisms enriched in diseased fish, particularly eukaryotic taxa including parasitic and protozoan lineages. However, unlike *F. psychrophilum*, these agents showed markedly inconsistent occurrence patterns across individuals. Some diseased fish were enriched for one eukaryotic taxon, whereas others carried different microorganisms, and none displayed the strong cross-individual recurrence observed for *F. psychrophilum*. Moreover, some were broadly distributed across multiple organs rather than being concentrated in the major lesion-associated tissues, such as skin and muscle, where pathological damage was most severe. Such patterns suggest that these microorganisms are less likely to represent the primary etiological driver of OWS. Instead, these organisms may reflect opportunistic or secondary infections that emerge following tissue damage, immune dysregulation, or barrier disruption caused by the primary bacterial infection. Similar ecological dynamics are commonly observed in aquatic disease systems, where an initial pathogen weakens host defenses and subsequently facilitates secondary colonization by fungi, parasites, or environmental microorganisms, thereby exacerbating tissue necrosis and disease severity (Dayana Senthamarai et al. 2023; Hou et al. 2023; O’Halloran et al. 2022; Okon et al. 2023; Rameshkumar et al. 2024). Importantly, our controlled infection experiments demonstrated that these additional microorganisms were not required to reproduce the disease phenotype. Experimental infection with *F. psychrophilum* alone successfully recapitulated the major clinical signs, histopathological lesions, mortality patterns, and infectome signatures of natural OWS. Nevertheless, the consistent enrichment of these secondary microorganisms in naturally diseased fish suggests that they may still contribute to disease progression under field conditions. Rather than acting as the primary cause of OWS, they may aggravate tissue destruction, accelerate host deterioration, or increase mortality once primary infection has been established. These findings further highlight the importance of distinguishing primary pathogens from secondary or opportunistic members of the infectome when interpreting metatranscriptomic data from complex disease systems.

Beyond resolving the etiology of OWS, our study also expands the known diversity of potential pathogens associated with grass carp. We identified several previously undescribed viral lineages, including the first hepacivirus reported from grass carp and a salmonid-associated nidovirus detected in a cyprinid host. Although these viruses were not prioritized as the primary drivers of OWS in the present cohort, their detection highlights the broad and largely unexplored reservoir of potential pathogens carried by intensively farmed fish. Such agents may remain clinically silent under current condition and host, but could become relevant under different host, environmental, or co-infection contexts. Therefore, total infectome analysis provides value beyond immediate etiological diagnosis: it can reveal latent or overlooked pathogen diversity that may contribute to future disease emergence.

**Fig. 6:**
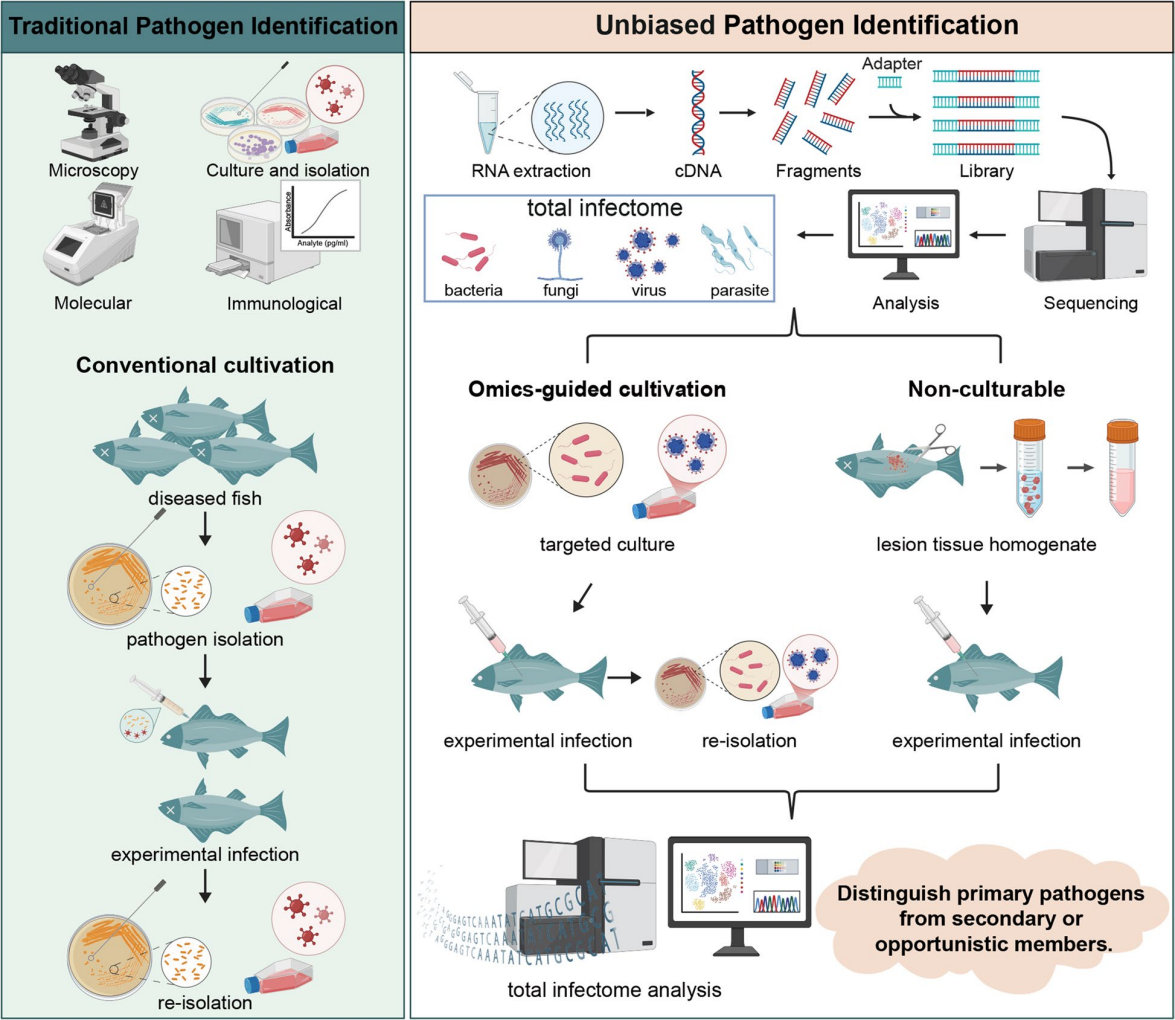
A total infectome-guided pathogen discovery and validation workflow for resolving complex disease etiology. The traditional pathogen identification strategy relies on pathogen isolation and experimental validation to fulfill Koch’s postulates. The unbiased pathogen identification framework integrates total infectome analysis with targeted isolation, experimental infection, re-isolation, and post-challenge infectome profiling

More importantly, this study establishes a generalizable framework for resolving complex disease etiology in systems characterized by polymicrobial backgrounds. Rather than relying solely on molecular detection or conventional isolation (Fig. 6), our strategy integrates epidemiological investigation, cohort-based comparative infectomics, targeted pathogen validation, and post-challenge infectome profiling into a coherent evidence chain linking pathogen discovery to causal inference. This framework enables prioritization of microorganisms that are consistently associated with disease while reducing the risk of misclassifying opportunistic or secondary microbes as primary pathogens. Notably, because the applicability of this framework depends heavily on factors such as cohort representativeness, sampling coverage, and the abundance and detectability of pathogen-derived transcriptional signals, sampling strategies and validation workflows must be rigorously tailored to the unique ecological and biological characteristics of each disease system. By incorporating such disease-specific optimization, this framework provides a flexible and transferable strategy for disentangling complex infectious etiologies across diverse biological contexts. Beyond aquaculture, such an approach may have broad applicability for emerging infectious diseases in wildlife, livestock, and clinical systems where complex microbial communities increasingly complicate etiological interpretation.

## Methods

### Epidemiological surveys and sampling

From 2021 to 2025, epidemiological surveys of grass carp OWS were conducted between January and April across multiple regions of China, including Chongqing, Guangdong, Henan, Hubei, Hunan, Jiangsu, Jiangxi, Ningxia, Shandong, Sichuan, and Zhejiang. Healthy and OWS-affected grass carp were collected from aquaculture farms. OWS-affected fish were identified based on characteristic clinical signs of OWS, such as external hemorrhages, subcutaneous muscle ulceration, reddening of the head, and swelling and erythema of the snout, as well as abnormal behavior (e.g., lethargy, reduced feeding activity, and aggregation near the pond edge), and were collected from ponds experiencing OWS outbreaks. Healthy fish exhibited normal swimming behavior and feeding activity, with no visible external lesions or other clinical signs of disease. Eight organs, including liver, spleen, kidney, intestine, gill, brain, muscle, and skin, were sampled from each fish, with muscle tissues collected from the margins of lesions. All tissue samples were immediately immersed in RNAlater™ (Invitrogen, Waltham, MA, USA), transported on dry ice, and stored at −80 °C until further processing.

### RNA extraction, library preparation and metatranscriptomic sequencing

Total RNA was automatically extracted and purified using the RNeasy 96 QIAcube HT Kit on the QIAGEN HT platform (Qiagen, Germany). RNA quality and concentration were assessed using a NanoDrop microvolume spectrophotometer and a Qubit fluorometer (Thermo Fisher Scientific, USA). Metatranscriptomic libraries were automatically prepared using the Ribo-Clean rRNA Depletion Kit Mega (AA) and the VAHTS Universal V8 RNA-seq Library Prep Kit for Illumina on the VNL-96P Automated Liquid Handling Workstation (Vazyme, China). Library quality and fragment size distribution were evaluated using the High Sensitivity D1000 ScreenTape® on the Agilent 4200 TapeStation system (Agilent Technologies, USA). Qualified libraries were sequenced on the Illumina NovaSeq 6000 platform to generate 150-bp paired-end reads.

### Identification of the fish host

To genetically confirm host identity, all available COI-5P sequences (the 5′ region of cytochrome c oxidase subunit I, COX1) of Actinopterygii were retrieved from the Barcode of Life Data System (BOLD) to construct a custom reference database comprising 318,438 sequences (Ratnasingham and Hebert 2007). For each library, COI-5P contigs were generated by mapping reads to the custom database and assembling the mapped reads. Maximum-likelihood phylogenetic trees were reconstructed using IQ-TREE (v1.6.12) with the parameters -m MFP -bb 1000 -bnni to screen putative COI-5P contigs(Nguyen et al. 2015). The selected COI-5P sequences were subsequently submitted to the BOLD platform for species-level identification.

### Metatranscriptomic data processing and pathogen discovery

Raw reads were subjected to quality control using Bbduk (Nabakooza et al. 2024) to remove adapter contamination, low-quality reads, and low-complexity regions. Duplicate reads were removed using cd-hit-dup (Fu et al. 2012), and rRNA reads were filtered by mapping against the SILVA rRNA database (Release 138.1) using Bowtie2 (v2.3.5.1) in local alignment mode (Langmead and Salzberg 2012; Quast et al. 2013). High-quality non-rRNA reads were then assembled de novo using MEGAHIT (v1.2.8) and annotated against the NCBI non-redundant (nr) protein database using DIAMOND BLASTx (e-value = 1 × 10^−5) (Buchfink et al. 2015; Li et al. 2015). Contigs assigned to viral taxa were extracted for downstream analysis.

For bacterial and eukaryotic microorganisms, custom reference databases were constructed using RNA polymerase beta subunit (rpoB) sequences retrieved from GTDB (release 214) and elongation factor 1 alpha (EF1α) sequences retrieved from NCBI. Candidate reads were identified by DIAMOND BLASTx against these reference databases and subsequently assembled using MEGAHIT. The resulting contigs were further annotated against the nr database using DIAMOND BLASTx (e-value = 1 × 10^−5) to infer their taxonomic and functional identities.

Contigs shorter than 450 bp were discarded, and overlapping contigs were merged into longer sequences using SnapGene (v8.1). Contigs showing amino acid identities below the defined thresholds relative to known species (viruses < 90%, bacteria < 95%, and eukaryotes < 97%) (Bondoso et al. 2013; Roger et al. 1999; Wang et al., 2023) were considered putative novel species. For phylogenetic analysis, viral replicase sequences (RNA viruses, RdRp domain(Venkataraman et al. 2018); DNA viruses, major capsid proteins or penton), bacterial rpoB, and eukaryotic EF1α sequences were aligned using MAFFT (v7.480; E-INS-i algorithm), and poorly aligned regions were removed using trimAl (v1.2) (Capella-Gutiérrez et al. 2009; Katoh et al. 2002). Maximum-likelihood phylogenies were then inferred using IQ-TREE under the LG amino acid substitution model.

### Estimation and comparative analysis of microorganism abundance

To estimate microbial abundance in each library, non-rRNA reads were mapped to viral, bacterial, and eukaryotic reference genomes using Bowtie2 with the –end-to-end and –very-fast parameters. Alignments were sorted and indexed using SAMtools (v1.18) (Li et al. 2009). To reduce false-positive assignments, assembled contigs were compared against the NCBI non-redundant nucleotide database using BLASTn, and sequences derived from the host genome, endogenous viral elements, or artificial vectors were removed. Microbial abundance was normalized as RPM, and only microorganisms with abundances > 1 RPM were retained (Doxey et al. 2025; Shi et al. 2022). The abundance matrix was log-transformed as log_10_ (x + 1) and visualized as a heatmap. Differential abundance between diseased (DGL) and healthy (HGL) groups was assessed after prevalence filtering, retaining microorganisms detected in at least eight samples in either group. Log_2_ fold change (log_2_FC) was calculated as log_2_ ((mean_DGL +0.001)/(mean_HGL +0.001)). Statistical significance was evaluated using the Wilcoxon rank-sum test with Benjamini–Hochberg false-discovery-rate (FDR) correction. Microorganisms with |log_2_FC|≥ 2 and FDR < 0.05 were considered significant. Volcano plots were generated using ggplot2 and ggrepel in R (version 4.4.2).

### Organ distribution analysis of *F. psychrophilum*

To assess the organ distribution of *F. psychrophilum* in healthy and diseased grass carp, microbial abundance was normalized as RPM and transformed as log_10_(RPM +1). Differences in *F. psychrophilum* abundance between healthy and diseased groups were evaluated separately for each organ using the Wilcoxon rank-sum test. Because tied values were present in the dataset, approximate *P* values were calculated. The transformed abundance values were visualized using boxplots.

### Bacterial isolation and taxonomic identification

Bacterial isolation was performed on oligotrophic TYES medium (Michel et al. 1999) and incubated at 15 °C for 72–96 h. Predominant colonies showing robust growth were selected and subcultured to obtain pure isolates. Taxonomic identification was conducted by amplifying and sequencing the 16S rRNA gene using the following primers: 27 F, 5′-AGAGTTTGATCMTGGCTCAG-3′; and 1492R, 5′-TACGGYTACCTTGTTACGACTT-3′ (Sabat et al. 2017). The resulting sequences were compared against the EZBioCloud database (Yoon et al. 2017) for species-level identification. Sequence similarity analysis and phylogenetic reconstruction were performed using MEGA12 (Kumar et al. 2024) with the neighbor-joining method. The identified isolates were preserved in glycerol stocks at −80 °C for future use.

### Challenge experiments

A total of 500 healthy grass carp (30 ± 5 cm in body length and 400 ± 50 g in body weight) were obtained from a fish farm in Shunde, Guangdong. Prior to the experiment, fish were acclimated in laboratory tanks for 1 week under continuous aeration with 30% daily water renewal. The rearing temperature was maintained at 26 °C, and fish were fed a commercial diet twice daily.

For bacterial challenge, *F. psychrophilum* GC30-154, isolated from lesion-associated muscle tissues of naturally diseased grass carp, was used. Fish were anesthetized with MS-222 and then injected intramuscularly into the dorsal musculature with graded doses of bacteria under different experimental conditions: (1) Dose-dependent infection at 15 °C: Fish were injected with 10^4, 10^5, 10^6, 10^7, and 10^8 CFU per fish. (2) Temperature-dependent infection at 10 °C, 15 °C, and 20 °C: Fish received 10^6 CFU per fish at each temperature to evaluate the effect of water temperature on disease progression. (3) Temperature shift experiment (10–15 °C): Fish were injected with 10^6 CFU per fish at 10 °C, followed by gradual warming to 15 °C, to assess the influence of temperature change on disease development.

PBS-injected fish served as negative controls. Following injection, fish were monitored daily for clinical signs and mortality. Moribund or deceased fish were immediately necropsied, and muscle samples were collected for bacterial re-isolation on TYES agar at 15 °C, followed by identification using 16S rRNA gene sequencing and phylogenetic analysis. This experimental design allowed evaluation of the pathogenicity of *F. psychrophilum* across different bacterial doses, rearing temperatures, and dynamic temperature conditions, enabling direct comparison with naturally occurring OWS in grass carp.

### Histopathological examination

Tissue samples from naturally diseased fish, experimentally infected fish, and their corresponding control groups were collected for histopathological analysis. The samples were fixed, routinely dehydrated through a graded ethanol series, embedded in paraffin, sectioned, and stained with hematoxylin and eosin (H&E). Histopathological changes were subsequently examined and recorded under a light microscope.

### Post-challenge total infectome analysis

To verify that *F. psychrophilum*, not secondary microbes, was the principal driver of disease in the experiment, post-challenge total infectome analysis was conducted. Samples from eight organs, including liver, spleen, kidney, intestine, gill, brain, muscle, and skin, were collected from *F. psychrophilum*-infected grass carp and PBS-injected control fish for total infectome analysis. Total RNA extraction, library preparation, sequencing, data processing, pathogen identification, and abundance estimation were performed as described above.

## Supplementary Information

Supplementary Material 1. Figure S1. Sample collection and metatranscriptomic sequencing in grass carp.(A). Workflow of sample collection, organ dissection, RNA extraction, library preparation, quality control, high-throughput sequencing, and bioinformatic analysis. Figure created with BioRender.com. (B). Proportions of remaining rRNA and non-rRNA reads across different organs after sequencing. (C). A phylogenetic tree based on the COI-5P gene of grass carp.

Supplementary Material 2. Figure S2. Phylogenetic trees of microorganisms detected in grass carp. (A). Bacterial phylogenetic tree inferred from rpoB protein sequences. (B). Eukaryotic phylogenetic tree inferred from EF1α protein sequences. (C). Viral phylogenetic trees constructed using RdRp for RNA viruses, penton for Orthopolintovirales, and major capsid protein for Petitvirales. Trees were reconstructed using the maximum likelihood method and midpoint-rooted, with branch lengths representing the number of amino acid substitutions per site. Dots indicate taxa detected in this study, with different colors representing the organs of origin.

Supplementary Material 3. Figure S3 Phylogenetic analysis of four novel RNA viruses and their abundance correlations with Ichthyobodonidae gen. sp.(A). Maximum likelihood phylogenetic tree of four novel RNA viruses identified in this study—Shunde narna-like virus 1, Shunde narna-like virus 2, Shunde tombus-like virus, and Shunde Bunya-Arena virus—based on RdRp sequences. Viruses are classified into Tombus-Noda, Narna, and Bunya-Arena viral groups. (B). Scatter plots showing significant positive correlations between the abundance of the four novel RNA viruses (Shunde narna-like virus 1, Shunde narna-like virus 2, Shunde tombus-like virus, and Shunde Bunya-Arena virus) and Ichthyobodonidae gen. sp. are shown, with Pearson correlation coefficients and p-values indicated.

Supplementary Material 4. Figure S4 Bacterial re-isolation and total infectome analysis after challenge. (A). Colony morphology of bacteria re-isolated from diseased fish after challenge in different injection-dose groups. (B). Heatmap of total infectome analysis after challenge, showing the abundance distribution of representative microorganisms across different organs in experimentally infected fish and PBS-injected control fish. The top annotations indicate sample type and organ type, and the color scale represents relative abundance.

## Data Availability

Raw sequencing reads are publicly available via the Intelligent Platform for Aquatic Animal Pathogens (https://aquapathognexus.cn/) under the project accession PRJOWS202606. All sequence alignments (fasta format), and phylogenetic trees (newick format) are available at 10.6084/m9.figshare.32881055.
