# Supplementary material for: A total infectome framework for resolving complex disease etiology in aquaculture
Source: Adv Biotechnol (Singap). 2026 Aug 7;4(3):31. doi: 10.1007/s44307-026-00125-8 (PMC13451469; doi:10.1007/s44307-026-00125-8)
Supplement: Supplementary file 1 [file 44307_2026_125_MOESM1_ESM.pdf]

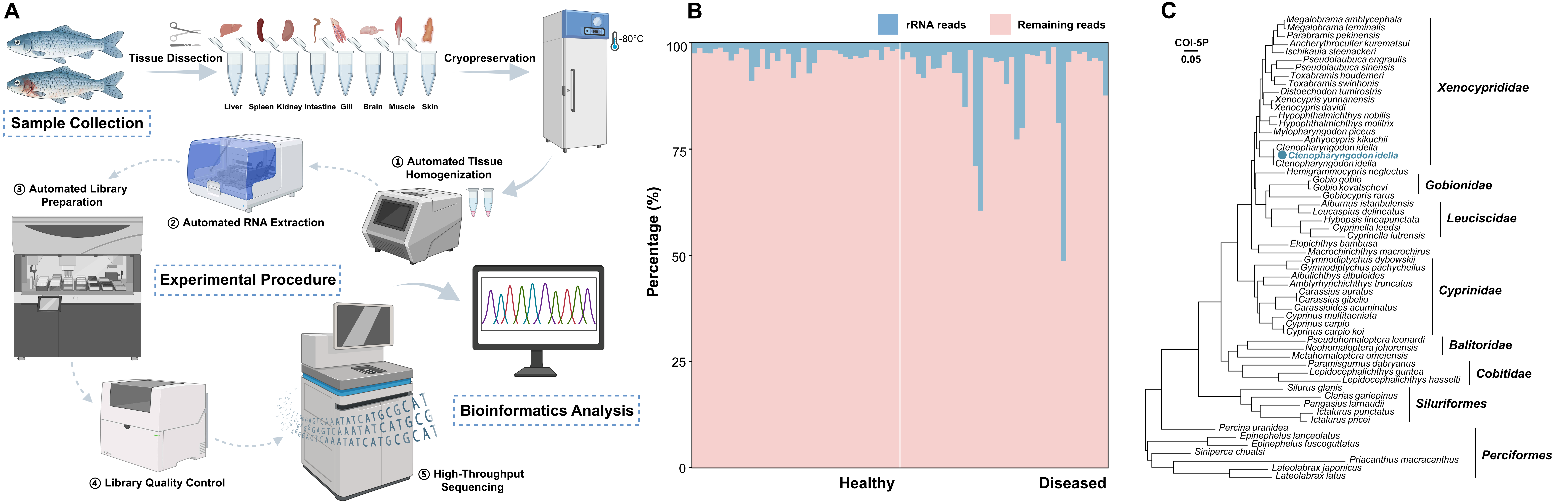
A
Tissue Dissection
Liver
Spleen
Kidney
Intestine
Gill
Brain
Muscle
Skin
Sample Collection
Cryopreservation
-80°C
① Automated Tissue Homogenization
② Automated RNA Extraction
③ Automated Library Preparation
Experimental Procedure
④ Library Quality Control
⑤ High-Throughput Sequencing
Bioinformatics Analysis
B
rRNA reads
Remaining reads
Percentage (%)
100
75
50
25
0
Healthy
Diseased
C
COI-5P
0.05
Megalobrama amblycephala
Megalobrama terminalis
Parabramis pekinensis
Ancherythroculter kurematsui
Ischikauia steenackeri
Pseudolaubuca engraulis
Pseudolaubuca sinensis
Toxabramis houdemeri
Toxabramis swinhonis
Distoechodon tumirostris
Xenocypris yunnanensis
Xenocypris davidi
Hypophthalmichthys nobilis
Hypophthalmichthys molitrix
Mylopharyngodon piceus
Aphyocypris kikuchii
Ctenopharyngodon idella
Ctenopharyngodon idella
Ctenopharyngodon idella
Xenocyprididae
Hemigrammocypris neglectus
Gobio gobio
Gobio kovatschevi
Gobiocypris rarus
Gobionidae
Alburnus istanbulensis
Leucaspius delineatus
Hybopsis lineapunctata
Cyprinella leedsi
Cyprinella lutrensis
Leuciscidae
Elopichthys bambusa
Macrochirichthys macrochirus
Gymnodiptychus dybowskii
Gymnodiptychus pachycheilus
Albulichthys albuloides
Amblyrhynchichthys truncatus
Carassius auratus
Carassius gibelio
Carassioides acuminatus
Cyprinus multitaeniata
Cyprinus carpio
Cyprinus carpio koi
Cyprinidae
Pseudohomaloptera leonardi
Neohomaloptera johorensis
Metahomaloptera omeiensis
Balitoridae
Paramisgurnus dabryanus
Lepidocephalichthys guntea
Lepidocephalichthys hasselti
Cobitidae
Silurus glanis
Clarias gariepinus
Pangasius larnaudii
Ictalurus punctatus
Ictalurus pricei
Siluriformes
Percina uranidea
Epinephelus lanceolatus
Epinephelus fuscoguttatus
Siniperca chuatsi
Priacanthus macracanthus
Lateolabrax japonicus
Lateolabrax latus
Perciformes
