# Supplementary material for: A total infectome framework for resolving complex disease etiology in aquaculture
Source: Adv Biotechnol (Singap). 2026 Aug 7;4(3):31. doi: 10.1007/s44307-026-00125-8 (PMC13451469; doi:10.1007/s44307-026-00125-8)
Supplement: Supplementary file 2 [file 44307_2026_125_MOESM2_ESM.pdf]

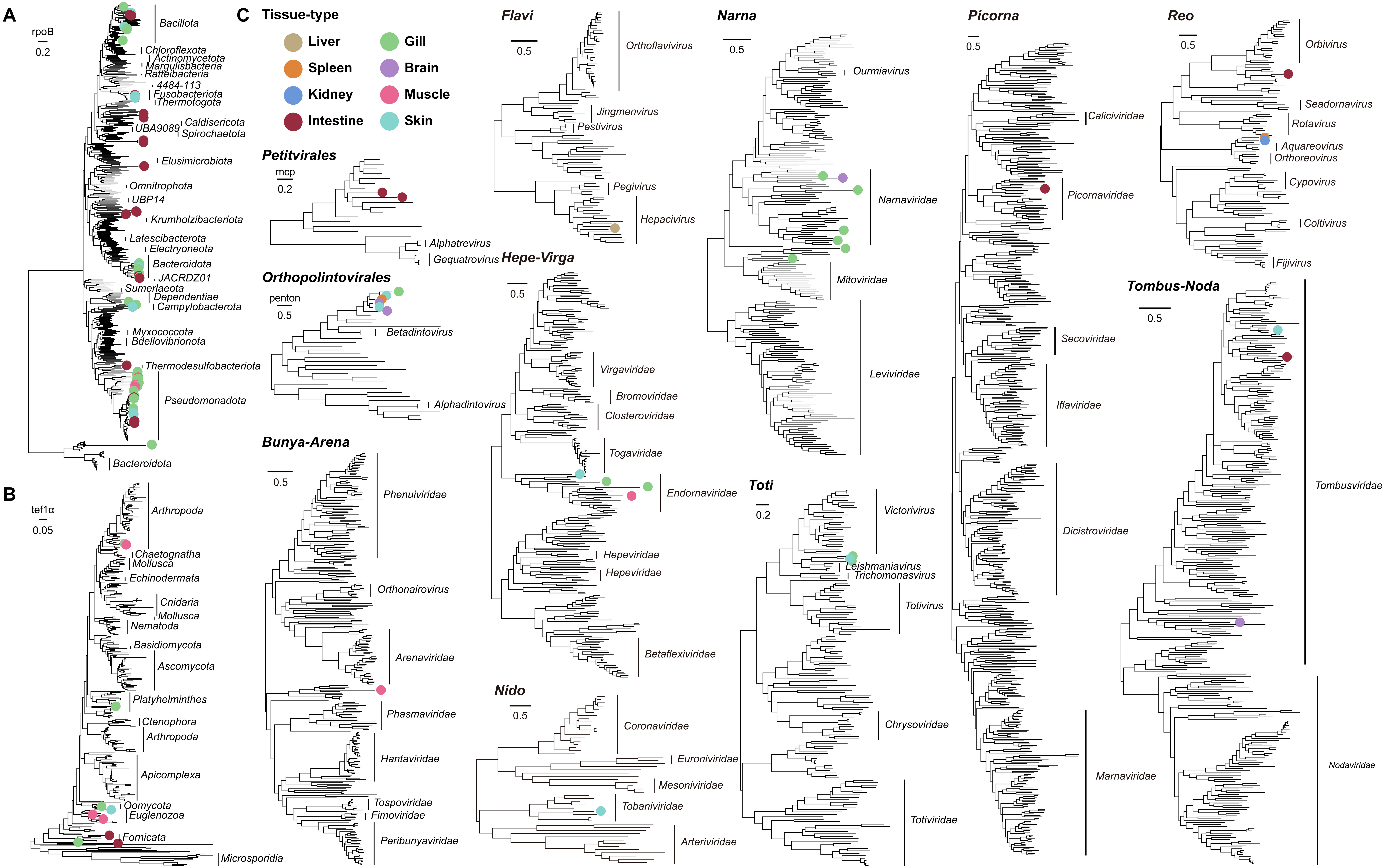
A
rpoB
0.2
Bacillota
Chloroflexota
Actinomycetota
Margulisbacteria
Ratteibacteria
4484-113
Fusobacteriota
Thermotogota
Caldisericota
UBA9089
Spirochaetota
Elusimicrobiota
Omnitrophota
UBP14
Krumholzibacteriota
Latescibacterota
Electryoneota
Bacteroidota
JACRDZ01
Sumerlaeota
Dependentiae
Campylobacterota
Myxococcota
Bdellovibrionota
Thermodesulfobacteriota
Pseudomonadota
Bacteroidota
B
tef1α
0.05
Arthropoda
Chaetognatha
Mollusca
Echinodermata
Cnidaria
Mollusca
Nematoda
Basidiomycota
Ascomycota
Platyhelminthes
Ctenophora
Arthropoda
Apicomplexa
Oomycota
Euglenozoa
Fornicata
Microsporidia
C
Tissue-type
Liver
Spleen
Kidney
Intestine
Gill
Brain
Muscle
Skin
Petitvirales
mcp
0.2
Alphatrevirus
Gequatrovirus
Orthopolintovirales
penton
0.5
Betadintovirus
Alphadintovirus
Bunya-Arena
0.5
Phenuiviridae
Orthonairovirus
Arenaviridae
Phasmaviridae
Hantaviridae
Tospoviridae
Fimoviridae
Peribunyaviridae
Flavi
0.5
Orthoflavivirus
Jingmenvirus
Pestivirus
Pegivirus
Hepacivirus
Hepe-Virga
0.5
Virgaviridae
Bromoviridae
Closteroviridae
Togaviridae
Endornaviridae
Hepeviridae
Hepeviridae
Betaflexiviridae
Nido
0.5
Coronaviridae
Euroniviridae
Mesoniviridae
Tobaniviridae
Arteriviridae
Narna
0.5
Ourmiavirus
Narnaviridae
Mitoviridae
Leviviridae
Toti
0.2
Victorivirus
Leishmaniavirus
Trichomonasvirus
Totivirus
Chrysoviridae
Totiviridae
Picorna
0.5
Caliciviridae
Picornaviridae
Secoviridae
Iflaviridae
Dicistroviridae
Marnaviridae
Reo
0.5
Orbivirus
Seadornavirus
Rotavirus
Aquareovirus
Orthoreovirus
Cypovirus
Coltivirus
Fijivirus
Tombus-Noda
0.5
Tombusviridae
Nodaviridae
