# Supplementary material for: A total infectome framework for resolving complex disease etiology in aquaculture
Source: Adv Biotechnol (Singap). 2026 Aug 7;4(3):31. doi: 10.1007/s44307-026-00125-8 (PMC13451469; doi:10.1007/s44307-026-00125-8)
Supplement: Supplementary file 3 [file 44307_2026_125_MOESM3_ESM.pdf]

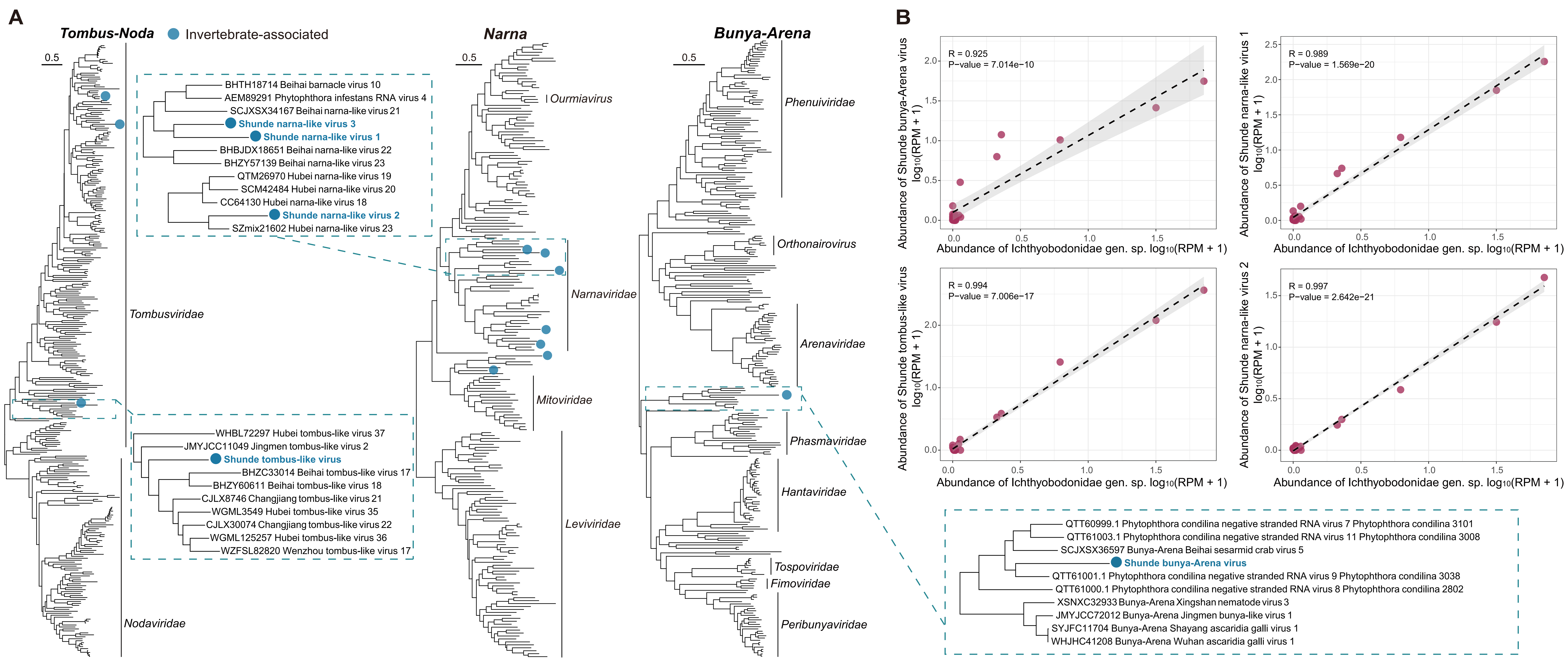

A
Tombus-Noda
Invertebrate-associated
0.5
BHTH18714 Beihai barnacle virus 10
AEM89291 Phytophthora infestans RNA virus 4
SCJXSX34167 Beihai narna-like virus 21
Shunde narna-like virus 3
Shunde narna-like virus 1
BHBJDX18651 Beihai narna-like virus 22
BHZY57139 Beihai narna-like virus 23
QTM26970 Hubei narna-like virus 19
SCM42484 Hubei narna-like virus 20
CC64130 Hubei narna-like virus 18
Shunde narna-like virus 2
SZmix21602 Hubei narna-like virus 23
Tombusviridae
WHBL72297 Hubei tombus-like virus 37
JMYJCC11049 Jingmen tombus-like virus 2
Shunde tombus-like virus
BHZC33014 Beihai tombus-like virus 17
BHZY60611 Beihai tombus-like virus 18
CJLX8746 Changjiang tombus-like virus 21
WGML3549 Hubei tombus-like virus 35
CJLX30074 Changjiang tombus-like virus 22
WGML125257 Hubei tombus-like virus 36
WZFSL82820 Wenzhou tombus-like virus 17
Nodaviridae
Narna
0.5
Ourmiavirus
Narnaviridae
Mitoviridae
Leviviridae
Bunya-Arena
0.5
Phenuiviridae
Orthonairovirus
Arenaviridae
Phasmaviridae
Hantaviridae
Tospoviridae
Fimoviridae
Peribunyaviridae
B
R = 0.925
P-value = 7.014e−10
Abundance of Shunde bunya-Arena virus log10(RPM + 1)
Abundance of Ichthyobodonidae gen. sp. log10(RPM + 1)
R = 0.989
P-value = 1.569e−20
Abundance of Shunde narna-like virus 1 log10(RPM + 1)
Abundance of Ichthyobodonidae gen. sp. log10(RPM + 1)
R = 0.994
P-value = 7.006e−17
Abundance of Shunde tombus-like virus log10(RPM + 1)
Abundance of Ichthyobodonidae gen. sp. log10(RPM + 1)
R = 0.997
P-value = 2.642e−21
Abundance of Shunde narna-like virus 2 log10(RPM + 1)
Abundance of Ichthyobodonidae gen. sp. log10(RPM + 1)
QTT60999.1 Phytophthora condilina negative stranded RNA virus 7 Phytophthora condilina 3101
QTT61003.1 Phytophthora condilina negative stranded RNA virus 11 Phytophthora condilina 3008
SCJXSX36597 Bunya-Arena Beihai sesarmid crab virus 5
Shunde bunya-Arena virus
QTT61001.1 Phytophthora condilina negative stranded RNA virus 9 Phytophthora condilina 3038
QTT61000.1 Phytophthora condilina negative stranded RNA virus 8 Phytophthora condilina 2802
XSNXC32933 Bunya-Arena Xingshan nematode virus 3
JMYJCC72012 Bunya-Arena Jingmen bunya-like virus 1
SYJFC11704 Bunya-Arena Shayang ascaridia galli virus 1
WHJHC41208 Bunya-Arena Wuhan ascaridia galli virus 1
