# Supplementary material for: A total infectome framework for resolving complex disease etiology in aquaculture
Source: Adv Biotechnol (Singap). 2026 Aug 7;4(3):31. doi: 10.1007/s44307-026-00125-8 (PMC13451469; doi:10.1007/s44307-026-00125-8)
Supplement: Supplementary file 4 [file 44307_2026_125_MOESM4_ESM.pdf]

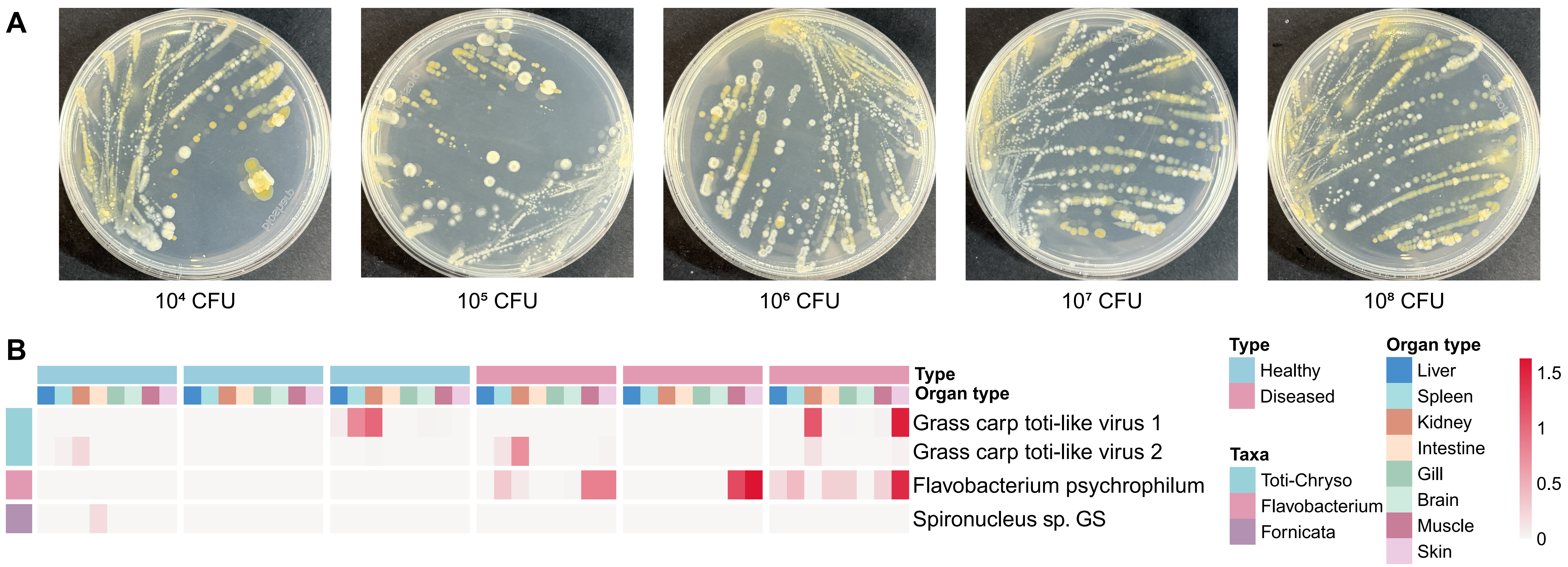
A
10⁴ CFU
10⁵ CFU
10⁶ CFU
10⁷ CFU
10⁸ CFU
B
Type
Organ type
Grass carp toti-like virus 1
Grass carp toti-like virus 2
Flavobacterium psychrophilum
Spironucleus sp. GS
Type
Healthy
Diseased
Taxa
Toti-Chryso
Flavobacterium
Fornicata
Organ type
Liver
Spleen
Kidney
Intestine
Gill
Brain
Muscle
Skin
1.5
1
0.5
0
